# Associations Between Decision-Making Biases and Swallowing and Physical Functions in Community-Dwelling Older Adults: A Cross-Sectional Study

**DOI:** 10.3390/geriatrics10060138

**Published:** 2025-10-24

**Authors:** Ayane Horike, Kohei Yamaguchi, Kanako Toda Shibahara, Jun Aida, Rieko Moritoyo, Kanako Yoshimi, Kazuharu Nakagawa, Haruka Tohara

**Affiliations:** 1 Department of Dysphagia Rehabilitation, Graduate School of Medical and Dental Sciences, Institute of Science Tokyo, 1-5-45 Yushima, Bunkyo-ku, Tokyo 113-8510, Japan; a.horike.swal@tmd.ac.jp (A.H.); r.moritoyo.swal@tmd.ac.jp (R.M.); k.yoshimi.gerd@tmd.ac.jp (K.Y.); nakagerd@tmd.ac.jp (K.N.); h.tohara.swal@tmd.ac.jp (H.T.); 2 Department of Health Sciences, Saitama Prefectural University, 820 Minomiya, Koshigaya-shi 343-8540, Saitama, Japan; shibahara-kanako@spu.ac.jp; 3 Department of Oral Health Promotion, Graduate School of Medical and Dental Sciences, Institute of Science Tokyo, 1-5-45 Yushima, Bunkyo-ku, Tokyo 113-8510, Japan; aida.j.e32f@m.isct.ac.jp

**Keywords:** decision-making biases, older adults, swallowing function, physical function

## Abstract

**Background/Objective:** In the context of global aging, maintaining daily habits such as adequate nutrition and regular exercise are essential to achieve healthy aging. Therefore, the preservation of swallowing and physical functions is fundamental. Jaw-opening force, an important swallowing function, is linked to physical function. Daily health behaviors are shaped by decision-making biases, which influence decision-making. Individuals with high procrastination tendencies may be less likely to engage in health-promoting behaviors, potentially leading to functional decline. While such tendencies are associated with general health behaviors, little is known about their associations with swallowing and physical functions among older adults. The objective of this study was to examine the associations between decision-making biases and swallowing and physical functions in community-dwelling older adults. **Methods:** A questionnaire survey was conducted to collect basic information and assess decision-making biases. The jaw-opening force (swallowing function) and grip strength (physical function) were measured. Associations of decision-making biases with jaw-opening force and grip strength were examined using multivariable linear regression analysis. We further conducted sex-stratified sensitivity analyses. **Results:** This cross-sectional study targeted 107 community-dwelling older adults. There was a significant negative association between procrastination tendency and jaw-opening force (B = −0.715, *p* = 0.005), and grip strength (B = −1.552, *p* = 0.003), indicating that individuals with a propensity for procrastination had lower jaw-opening force and grip strength. **Conclusions:** Procrastination tendency may be used as an indicator to detect swallowing and physical functions. Moreover, incorporating this modifiable factor to promote behavior change may prevent functional decline. The study results highlight the significance of considering individuals’ decision-making biases—particularly procrastination tendency—in clinical settings.

## 1. Introduction

In the context of an increasingly aging population, the promotion of healthy aging has become a global priority. Achieving healthy aging requires maintaining daily habits, including proper nutrition and regular exercise, which influence swallowing and physical functions via muscle mass/quality [1]. In this regard, the preservation of both swallowing and physical functions is fundamental [2]. Swallowing-function-related abilities such as jaw-opening force play a vital role and have been reported to be associated with physical function [3]. However, advancing age is often accompanied by a gradual decline in functional abilities, which can lead to reduced food intake, malnutrition, frailty, and a subsequent deterioration in overall health and independence [4,5]. Therefore, the early identification of declines in both swallowing and physical functions is critical for supporting healthy aging in community-dwelling older adults.

Daily habits are a series of continuous decision-making activities that are influenced by individual’s decision-making biases [6]. Behavioral economics analyzes these human decision-making patterns and has highlighted time preference as a prominent factor [6,7]. Time preference includes the time discounting rate and present bias, characterized by a psychological tendency to prioritize immediate rewards over future benefits, often resulting in procrastination [8]. Individuals with a high propensity for procrastination tend to have difficulties engaging in health-promoting behaviors, such as maintaining a healthy diet or exercising regularly [9]. For instance, they are more likely to smoke, have a higher body mass index, and develop chronic diseases such as hypertension and cardiovascular diseases [10,11].

However, while associations between decision-making biases, including procrastination tendencies, and general health-related behaviors have been examined, little is known about how these traits are associated with swallowing and physical functions in older adults. The accumulation of unhealthy behaviors owing to decision-making biases may contribute to a decline in swallowing and physical functions. This explanatory study, which adopts a clinical perspective, aimed to investigate associations between decision-making biases and swallowing and physical functions among community-dwelling older adults. The elucidation of these associations could offer new insights into the development of preventive strategies for functional decline, considering individual differences in decision-making patterns to better support healthy aging.

## 2. Materials and Methods

### 2.1. Research Design and Settings

This cross-sectional study targeted community-dwelling older adults who were members of a senior club in Yokohama City, Kanagawa Prefecture, Japan. Senior clubs are volunteer groups that participate in various health projects aimed at contributing to society. Measurements were conducted between November 2022 to May 2025. The dataset used in this study partially overlapped with that of a previously published study [12], although additional data were included in the present analysis. Because this was a secondary analysis, no specific sample-size calculation was performed.

### 2.2. Participants

The inclusion criteria for this study included being community-dwelling adults aged 65 years or older who belong to the senior club in Yokohama City and who attended the measurement sessions conducted by researchers. Individuals who had significant difficulty in following the investigator’s instructions were excluded from the study.

### 2.3. Decision-Making Biases

In this study, we use “decision-making biases” as an umbrella term for three a priori constructs examined: procrastination tendency, time discounting, and risk preference. Questions on attitudes towards risk were included alongside those on procrastination and time discounting tendencies because risk-averse individuals are generally reported to be less likely to engage in risky behaviors, including smoking, excessive drinking, and unhealthy eating [13]. Decision-making biases were investigated based on questions selected from the Global Preference Survey (GPS) [14], an internationally surveyed dataset of decision-making biases. We used a modified, brief version of the GPS to reduce respondent burden while preserving validity, as supported by prior studies [13]. These items jointly capture (i) behavioral implementation barriers in daily self-care (procrastination), (ii) willingness to incur present costs for future health benefits (time discounting), and (iii) attitudes toward uncertainty in health-related choices (risk preference). To ensure cross-cultural validity, Japanese versions were created through forward–back translation by bilingual translators and were pilot-tested among older adults [13].

Responses to questions on decision-making biases (procrastination tendency, time discounting tendency, and attitude towards risk) were rated on an 11-point Likert scale with scores ranging from 0–10. Procrastination tendency was assessed by the following question: “Generally speaking, how often do you procrastinate on tasks that you know should be done right away? Please select the number that best describes you, with 0 meaning “not at all” and 10 meaning “very often.” Time discounting tendency was assessed using the following question: “Generally speaking, to what extent are you willing to give up something that benefits you now for a greater benefit in the future? Please select the number that best describes you, with 0 meaning “very willing to give up” and 10 meaning “not willing to give up at all.” Attitude towards risk was assessed using the following question: “Generally speaking, how willing are you to take risks? Please select the number that best describes you, with 0 meaning “very willing to take risks” and 10 meaning “not willing to take any risks at all.” To minimize bias, participants were asked to complete the questionnaire independently.

### 2.4. Outcomes

Jaw-opening force was assessed as an indicator of swallowing function [15] and is a simple screening tool for assessing swallowing function [16]; it reflects the strength and mobility of the suprahyoid muscles [17]. Jaw-opening force was measured using a jaw-opening sthenometer (Livet Inc., Tokyo, Japan). Participants were instructed to sit and relax with their mouths closed and to clench their teeth lightly. An adjustment belt was placed above the head, and a jaw cap was positioned below the jaw. Both were securely fixed to ensure accurate measurements. Participants were instructed to open their mouths as wide as possible. Jaw-opening force was measured three times for each participant, and the highest value was recorded.

Grip strength was assessed as an indicator of physical function [18]. Grip strength was measured twice in the dominant hand using a grip dynamometer (Toei Light Ltd., Saitama, Japan) in a standing posture, and the highest value was recorded.

All assessments were conducted by experienced dentists and physical therapists. To minimize inter-examiner discrepancies, each survey was conducted by the same staff member.

### 2.5. Covariates

The questionnaire collected demographic and health-related information, including participants’ age, sex, financial status, history of hypertension, and smoking status. Financial status was included as a covariate because socioeconomic factors have been shown to influence health behaviors and physical functioning in older adults [19]. Controlling for economic status allowed us to examine the independent association between decision-making biases and functional outcomes more accurately. We asked the following question: “Imagine you are having lunch alone on a weekday. How much are you willing to spend at most when you decide to ‘treat yourself’ a little?” This question had nine choices, ranging from <499 yen to >15,000 yen [12]. The history of hypertension and smoking status were self-reported as yes/no. The questionnaire was mailed to the participants in advance, and the investigators checked for missing entries on the day of the swallowing and physical function measurements.

### 2.6. Statistical Analysis

Descriptive statistics, Spearman’s rank correlation analysis, univariable and multivariable linear regression analysis were used to examine the association between decision-making biases and jaw-opening force, grip strength. Spearman’s rank correlation coefficient was calculated to examine the correlation between age, the three decision-making biases, and the financial status.

In the univariable linear regression analyses, jaw-opening force and grip strength were the dependent variables, and age, sex, the three decision-making biases, financial status, and hypertension were each entered separately as explanatory variables. In the multivariable linear regression analyses, jaw-opening force and grip strength were the dependent variables, and age, sex, the three decision-making biases, financial status, and hypertension were entered simultaneously as explanatory variables. All models used the forced-entry method, with sex coded as a binary variable (1 = male, 2 = female) and hypertension coded as a binary indicator (1 = hypertension, 0 = no hypertension). To standardize the data, each of the three decision-making biases was converted to a Z-score (mean = 0, standard deviation = 1). Analyses were conducted using the Japanese version of SPSS for Windows (Version 28; IBM Japan, Ltd., Tokyo, Japan).

## 3. Results

### 3.1. Characteristics of Participants

In total, 107 people participated in the study. Following the exclusion of 8 participants who could not complete the questionnaire survey and 1 person who declined to participate, the final number of participants was 98. The data analysis of jaw-opening force included all 98 participants (37 men and 61 women; mean age, 79.4 ± 5.3 years; Table 1). Because data from 2 participants were missing, the data analysis of grip strength included 96 participants (36 men and 60 women).

Table 1 presents the descriptive survey results. The mean scores for the decision-making biases were as follows: procrastination tendency, 3.8 ± 2.5; time discounting tendency, 5.6 ± 3.0; and attitude towards risk, 6.5 ± 2.3. The mean score for the financial status was 4.2 ± 1.5. The mean jaw-opening force was 6.9 ± 2.6 kg, and the mean grip strength was 23.7 ± 6.5 kg. Among the participants, 39.8% had hypertension, and 25.3% reported a history of smoking.

### 3.2. Correlations Among Decision-Making Biases and Covariates

The correlations for each variable are shown in Table 2. Among the examined decision-making biases, some showed notable intercorrelations. The strongest correlation was observed between the time discounting tendency and risk preference, with a significant positive correlation (r = 0.470, *p* < 0.01), suggesting that individuals with a lower time discounting tendency were more risk seeking.

### 3.3. Associations Between Decision-Making Biases and Jaw-Opening Force

Associations between the decision-making biases and jaw-opening force are shown in Table 3. A significant negative association was found between procrastination tendency and jaw-opening force, indicating that individuals with a propensity for procrastination had a lower jaw-opening force (B (95% confidence interval [CI]) = −0.720 (−1.222 to −0.219), *p* = 0.005). In addition, no significant association were observed between time discounting tendency, risk preference, and jaw-opening force.

### 3.4. Associations Between Decision-Making Biases and Grip Strength

Associations between the decision-making biases and grip strength are shown in Table 4. Individuals with a propensity for procrastination also had lower grip strength (B (95% CI) = −1.389 (−2.318 to −0.460), *p* = 0.004). No significant association were observed between time discounting tendency, risk preference, and grip strength.

## 4. Discussion

To the best of our knowledge, this is the first study to investigate the associations between decision-making biases and swallowing and physical functions among community-dwelling older adults. In this cross-sectional study, individuals with a higher tendency toward procrastination exhibited lower jaw-opening force and grip strength.

Previous studies have shown that procrastination tendency is associated with unhealthy behaviors such as poor nutritional practices and smoking [8,20]. Individuals with a high tendency to procrastinate are more present-oriented and are less likely to engage in health-promoting behaviors. Such behaviors, including inadequate fruit and vegetable intake, smoking and physical inactivity, have been linked to reduced functional capacity [21]. Individuals with a high procrastination tendency may experience functional decline due to the accumulation of unhealthy behaviors. Additionally, children with higher procrastination tendencies have been reported to have fewer teeth in old age [22]; furthermore, having fewer than 20 teeth is associated with a higher risk of mortality and functional disability later in life [23], suggesting that oral health mediates the association between procrastination tendency and functional decline. In this study, participants generally exhibited low procrastination tendencies, moderate time discounting, and were risk-averse (Table 1). These patterns align with GPS trends and prior research showing that older adults tend to procrastinate less, discount the future more steeply, and become more risk-averse with age [14,24,25,26]. Moreover, the positive correlation observed between lower time discounting tendency and greater risk-seeking (Table 2) is consistent with previous findings that patience is associated with increased risk-taking [14].

Physical activity levels may serve as a mediating factor in the association between procrastination tendency and both swallowing and physical functions. Jaw-opening force has been associated with physical activity levels, and decreased physical activity has been shown to reduce swallowing function [27]. Moreover, physical inactivity increases the risk of chronic diseases such as cardiovascular disease and stroke [28], yet approximately 31% of the global population fails to meet the recommended levels of physical activity [28]. Individuals with a strong present bias, a key component of procrastination, tend to prioritize immediate comfort over future health benefits and are less likely to engage in regular exercise [8]. Given the established associations between physical activity levels and swallowing function as well as those between procrastination tendency and physical activity levels, it is plausible that procrastination tendency contributes to the decline in swallowing function through reduced activity. Furthermore, jaw-opening force and grip strength have been reported to correlate with each other [29], and physical activity levels are also closely linked to physical function [30]. These findings support the possibility that procrastination tendency affects both swallowing and physical functions, via physical inactivity. Additionally, since sarcopenia is closely related to both swallowing and physical function in older adults, declines in swallowing and physical function may increase sarcopenia risk.

This study’s findings highlight the potential utility of assessing decision-making biases, particularly procrastination tendency, as indicators of swallowing and physical functions in clinical settings. Procrastination tendency may serve as a practical screening tool for identifying individuals at risk of functional decline. To address this tendency, behaviorally informed strategies, including “nudges,” may be effective. At the individual level, commitment devices—such as declaring daily swallowing exercise goals to clinicians—can increase accountability and reduce procrastination [31]. At the group level, social norm-based nudges—including communicating that “most older adults in your community complete their exercises”—may promote participation by leveraging peer influence [32]. These approaches, which encourage small but consistent behavior changes without coercion, align with behavioral economic principles and may contribute to the maintenance of swallowing and physical functions, supporting healthier aging and improved quality of life.

This study had some limitations. First, its cross-sectional design and modest sample size limit causal inference and statistical power; in particular, the sex-stratified analyses of jaw-opening force and grip strength were underpowered due to small within-sex samples and should be regarded as exploratory (see Supplementary File for details). Additionally, because participants were community-dwelling older adults who were independent in activities of daily living (ADL), medical history was obtained via a self-administered questionnaire rather than clinician-led interviews, which may have introduced residual confounding; future studies should broaden the target population beyond ADL-independent older adults and incorporate a comprehensive comorbidity assessment. Moreover, we did not collect direct measures of physical activity; therefore, the hypothesized pathway suggesting that procrastination leads to lower activity and, in turn, to reduced swallowing and physical functions remains plausible but untested. Future research should include validated physical-activity measures to examine this pathway empirically and assess mediation. Secondly, the decision-making biases in this study were assessed using self-reported Likert scale measures. While quantitative monetary-based hypothetical transactions are generally applied to assess decision-making biases, such approaches are susceptible to measurement errors, and are time-consuming in clinical settings, particularly in older adult patients. Given that biases can also be assessed through qualitative evaluation methods, we opted for a more straightforward approach. Moreover, in older adults, brief Likert-type measures have demonstrated acceptable validity; for example, simple pain intensity scales (e.g., NRS vs. VAS/VRS) show good validity and usability [33]. Although these tools are not gold standards, this evidence supports our pragmatic choice to reduce the respondent burden. In the future, the validation of the survey methods used to assess decision-making biases is necessary. As a third limitation, participants were self-selected volunteers who were likely to be more health-conscious than the general older adult population, which may limit the generalizability of the results. Nonetheless, as noted in the Discussion, the central tendency of our decision-making bias scores was comparable to published norms in similar age groups, partially mitigating concerns about external validity. Finally, dysphagia in older adults cannot be explained by muscle weakness alone; age-related sensory decline and cortical changes also contribute. As our study focused on strength, these factors were not captured. Future research should incorporate sensory and neurocognitive assessments to more comprehensively elucidate mechanisms of swallowing and physical function decline.

Despite these limitations, this study reveals an association between decision-making biases and swallowing and physical functions, offering practical insights for clinical and public-health strategies. While combining nudges with monetary incentives can enhance behavioral changes, limited resources make broad implementation challenging. Identifying procrastination tendency as a trait linked to functional decline may support more targeted and cost-effective interventions [34].

## 5. Conclusions

A propensity for procrastination was associated with lower jaw-opening force and grip strength. Assessing individuals’ procrastination tendency in clinical settings may be useful for detecting swallowing and physical functions.

## Figures and Tables

**Table 1:** Descriptive characteristics of participants (N = 98).

	Total (N = 98)	Range
Procrastination, points	3.8 ± 2.5	0–10
Time discounting, points	5.6 ± 3.0	0–10
Risk preference, points	6.5 ± 2.3	0–10
Age, years	79.4 ± 5.3	66–91
Sex, *n* (%)	98 (Men: 37.8%)	
Jaw-opening force, kg	6.9 ± 2.6	0.6–14.0
Grip strength, kg (*n* = 96)	23.7 ± 6.5	10.2–41.0
Financial status, points	4.2 ± 1.5	1–9
History of hypertension, *n* (%)	39 (39.8%)	
Smoking history, *n* (%) (*n* = 95)	24 (25.3%)	

Note: A high score for procrastination and time discounting indicates higher levels of procrastination and time discounting tendencies. A low score for risk preference indicates risk-seeking attitudes.

**Table 2:** Correlation coefficients among age, decision-making biases, and financial status (N = 98).

	Age	Procrastination	Time Discounting	Risk Preference	Financial Status
Age	1.000	0.000	0.096	0.042	−0.074
Procrastination		1.000	−0.200 *	−0.385 **	−0.094
Time discounting			1.000	0.469 **	−0.117
Risk preference				1.000	0.079
Financial status					1.000

* Correlation coefficient is significant at the 5% level. ** Correlation coefficient is significant at the 1% level. Note: A high score for procrastination and time discounting indicates higher levels of procrastination and time discounting tendencies. A low score for risk preference indicates risk-seeking attitudes.

**Table 3:** Associations between decision-making biases and jaw-opening force in the univariable and multivariable linear regression analyses (N = 98).

	Univariable Model					Multivariable Model				
	B	95% CI		*p*	Adjusted R^2^	B	95% CI		*p*	Adjusted R^2^
Age	−0.115	−0.209	−0.021	0.017	0.048	−0.132	−0.219	−0.045	0.003	0.205
Sex	−1.718	−2.728	−0.707	0.001	0.097	−2.116	−3.095	−1.138	*p* < 0.001	
Z score (procrastination)	−0.422	−0.936	0.092	0.106	0.017	−0.720	−1.222	−0.219	0.005	
Z score (time discounting)	−0.115	−0.635	0.405	0.661	−0.008	0.058	−0.472	0.588	0.828	
Z score (risk preference)	−0.232	−0.750	0.287	0.377	−0.002	−0.400	−0.953	0.153	0.154	
Financial status	0.117	−0.230	0.464	0.504	−0.006	0.167	−0.150	0.485	0.298	
Hypertension	−0.044	−1.103	1.014	0.934	−0.010	−0.045	−1.004	0.915	0.926	

Abbreviations: B, unstandardized coefficient; CI, confidence interval; *p*, *p*-value. Note: A high score on procrastination, time discounting indicates higher level of procrastination tendency and time discounting tendency. A low score on risk preference indicates risk-seeking attitudes.

**Table 4:** Associations between decision-making biases and grip strength in the univariable and multivariable linear regression analyses (N = 96).

	Univariable Model					Multivariable Model				
	B	95% CI		*p*	Adjusted R^2^	B	95% CI		*p*	Adjusted R^2^
Age	−0.247	−0.493	−0.001	0.049	0.030	−0.335	−0.501	−0.169	*p* < 0.001	0.574
Sex	−9.163	−11.184	−7.141	*p* < 0.001	0.457	−10.162	−12.004	−8.319	*p* < 0.001	
Z score (Procrastination)	−0.597	−1.933	0.740	0.378	−0.002	−1.389	−2.318	−0.460	0.004	
Z score (Time discounting)	0.168	−1.174	1.510	0.804	−0.010	0.628	−0.374	1.631	0.216	
Z score (Risk preference)	−0.393	−1.733	0.947	0.562	−0.007	−0.820	−1.852	0.213	0.118	
Financial status	0.339	−0.662	1.339	0.503	−0.006	0.624	−0.042	1.290	0.066	
Hypertension	0.228	−2.502	2.958	0.869	−0.010	−0.609	−2.422	1.204	0.506	

Abbreviations: B, unstandardized coefficient; CI, confidence interval; *p*, *p*-value. Note: A high score on procrastination, time discounting indicates higher level of procrastination tendency and time discounting tendency. We further conducted sex-stratified sensitivity analyses for jaw-opening force and grip strength; details are provided in the Supplementary File. A low score on risk preference indicates risk-seeking attitudes.

## Data Availability

The data supporting the findings of this study are not publicly available due to privacy concerns regarding research participants. However, they are available from the corresponding author upon reasonable request.

## Supplementary Materials

The following supporting information can be downloaded at: https://www.mdpi.com/article/10.3390/geriatrics10060138/s1, Table S1: Results of the sex-stratified sensitivity analyses for jaw-opening force (multivariable model); Table S2: Results of the sex-stratified sensitivity analyses for grip strength (multivariable model).

## Abbreviations

The following abbreviations are used in this manuscript:

GPS	Global Preference Survey
CL	Confidence interval
ADL	Activities of daily living
