# Supplementary material for: Associations Between Decision-Making Biases and Swallowing and Physical Functions in Community-Dwelling Older Adults: A Cross-Sectional Study
Source: Geriatrics (Basel). 2025 Oct 24;10(6):138. doi: 10.3390/geriatrics10060138 (PMC12641726; doi:10.3390/geriatrics10060138)
Supplement: Supplementary file 1 [file geriatrics-10-00138-s001.zip › geriatrics-3883273-supplementary.pdf]

Supplementary Analyses

We conducted a sex-stratified multivariable regression analyses, with jaw-opening force and grip strength as dependent variables.
For jaw-opening force, a significant association with procrastination tendency was observed in women (n=61), but not in men (n=38).
For grip strength, the overall regression model was not significant in women (n = 60; F = 2.385, p = 0.050), indicating that the model as a whole did not adequately explain the dependent variable. Nevertheless, exploratory analyses indicated a significant association between procrastination tendency and grip strength (β = -0.333, p = 0.011). Given the nonsignificant overall model, this finding should be considered tentative. In men (n = 37), no significant associations were observed.
The results are presented in Supplementary Tables 1 and 2.

Supplementary Table S1. Results of the sex-stratified sensitivity analyses for jaw-opening force (multivariable model)

	Men (n=38)					Women (n=61)				
	B	95%CI		p	Adjusted R^2	B	95%CI		p	Adjusted R^2
Age	-0.125	-0.285	0.035	0.121		-0.148	-0.255	-0.042	0.007	
Z score (Procrastination)	-1.029	-2.108	0.051	0.061		-0.592	-1.158	-0.026	0.041	
Z score (Time discounting)	0.009	-1.005	1.024	0.985	0.036	0.012	-0.629	0.654	0.969	0.130
Z score (Risk preference)	-0.723	-1.819	0.373	0.188		-0.214	-0.867	0.440	0.515	
Economic indicators	0.167	-0.494	0.828	0.611		0.144	-0.229	0.516	0.443	

Abbreviations: B, unstandardized coefficient; CI, confidence interval; p, p-value

Note: A high score on procrastination, time discounting indicates higher level of procrastination tendency and time discounting tendency.
A low score on risk preference indicates risk-seeking attitudes.

Supplementary Table S2. Results of the sex-stratified sensitivity analyses for grip strength (multivariable model)

	Men (n=37)					Women (n=60)				
	B	95%CI		p	Adjusted R^2	B	95%CI		p	Adjusted R^2
Age	-0.398	-0.691	-0.105	0.009		-0.210	-0.463	0.043	0.101	
Z score (Procrastination)	-1.364	-3.226	0.497	0.145		-1.733	-3.043	-0.422	0.011	
Z score (Time discounting)	0.834	-0.904	2.572	0.335	0.240	0.777	-0.766	2.319	0.317	0.105
Z score (Risk preference)	-1.073	-2.926	0.779	0.246		-0.904	-2.452	0.644	0.247	
Economic indicators	1.249	-0.185	2.683	0.086		0.377	-0.530	1.284	0.408	

Abbreviations: B, unstandardized coefficient; CI, confidence interval; p, p-value

Note: A high score on procrastination, time discounting indicates higher level of procrastination tendency and time discounting tendency.

A low score on risk preference indicates risk-seeking attitudes.
